# *Brucella*, *Coxiella*, and *Theileria* Species DNA in *Haemaphysalis qinghaiensis* Ticks Collected from Goats and Sheep in Qinghai Province, Northwest China

**DOI:** 10.3390/tropicalmed11010017

**Published:** 2026-01-07

**Authors:** Kun Li, Xuxin Yang, Jianling Wang, Shengyu Li, Xu Zhao, Shengjun Cai, Leyu Wu, Guoqiang An, Hongyan Zhao, Dongri Piao, Qingqing Xu, Yu Fan, Jiquan Li, Hai Jiang

**Affiliations:** 1 National Key Laboratory of Intelligent Tracing and Forecasting for Infectious Diseases, National Institute for Communicable Disease Control and Prevention, Chinese Center for Disease Control and Prevention, Changping District, Beijing 102206, China; 2 Qinghai Institute for Endemic Disease Prevention and Control, Xining 810021, China; 3 Daxing Center for Disease Control and Prevention, Daxing District, Beijing 102600, China; 4 Menyuan Center for Disease Control and Prevention, Haibei Tibetan Autonomous Prefecture 810399, China

**Keywords:** Qinghai Province, *Haemaphysalis qinghaiensis*, tick-borne pathogens, molecular detection, livestock, public health, PCR

## Abstract

*Haemaphysalis qinghaiensis* is an endemic tick species distributed in the western plateau areas of China. Although they are three-host ticks, infesting multiple animals (including humans), the occurrence of various tick-borne agents has barely been investigated. In this study, we collected 136 *H. qinghaiensis* specimens from sheep and goats in Menyuan County in Qinghai Province, northwest China. The *Brucella*, *Coxiella*, and *Theileria/Babesia* species’ DNA were detected by nested or hemi-nested PCR and further identified by amplifying their key genes. *Brucella abortus* and *B. melitensis* DNA were detected, with positive rates of 3.68% and 4.41%, respectively. This may be the first report that suggests that *H. qinghaiensis* harbors *Brucella* spp., the agents of human brucellosis. The *Coxiella* endosymbiont of *Haemaphysalis qinghaiensis*, a non-pathogenic *Coxiella,* was identified with an extremely high positive rate of 97.06%. In addition, two *Theileria* species, *Theileria luwenshuni* (75.00%) and *Theileria uilenbergi* (16.18%), were detected. Our results suggest the circulation of *Brucella* spp. and *Theileria* spp. in goats and sheep in the study area. Whether *H. qinghaiensis* ticks play a role in the maintenance and transmission of these agents has yet to be determined. Due to their human pathogenicity and their high positive rates in ticks, surveillance in local populations with relative symptoms is necessary.

## 1. Introduction

Tick-borne pathogens have been a global health concern in recent years. As the primary vectors for animal pathogens and the second vectors for human pathogens, ticks (Acari: Ixodida) include 896 species and are globally distributed [1]. They play a key role in the transmission/conservation of numerous human and animal pathogens, such as *Borrelia burgdorferi* sensu lato, *Coxiella burnetii*, *Rickettsia* spp., tick-borne encephalitis virus, severe fever with thrombocytopenia syndrome virus, etc. [2]. It has been reported that tick-borne pathogens are responsible for more than 100,000 human cases worldwide, posing a great threat to public health [3].

*Haemaphysalis qinghaiensis* Teng, 1980 (genus *Haemaphysalis*, family Ixodidae) is endemic to China. It is widely distributed in the western plateau of China, including the Qinghai, Gansu, and Sichuan provinces [4]. It usually infests domestic animals (such as sheep, goats, yaks, and cattle) and wild animals (such as hares) [5]. Occasionally, it also bites humans. As a three-host tick, it switches hosts during its development, thus possibly transmitting multiple pathogens between animals and humans. Many studies have been performed on *H. qinghaiensis*-vectored pathogens in China [5,6,7,8]. It has been reported that *H. qinghaiensis* harbors a large variety of pathogens of humans and animals, including spotted fever group *Rickettsia*, *Anaplasma bovis*, *Anaplasma phagocytophilum*, *Borrelia burgdorferi* sensu lato, *Theileria sinensis*, and *Theileria uilenbergi* [5,6,7,8].

Despite the wide distribution of *H. qinghaiensis* in China and its high prevalence in certain areas, studies on the tick-borne pathogens harbored by *H. qinghaiensis* are still relatively rare. In fact, the role of this species as a vector is still somewhat neglected. Meanwhile, some pathogens are rarely investigated in this tick species in most areas. Of those, *Brucella*, *Coxiella*, and *Theileria* are zoonotic pathogens widely circulated in northwest China [9]. They pose a great threat to husbandry, result in remarkable economic losses, and are becoming a risk to public health. However, to date, these pathogens are still rarely reported in *H. qinghaiensis* in China.

The aim of this study is to investigate the prevalence and perform genetic characterization of *Brucella*, *Coxiella*, and *Theileria* species in *Haemaphysalis qinghaiensis* ticks collected from Qinghai Province, China, to better understand their role as carriers of zoonotic and veterinary pathogens.

## 2. Methods

### 2.1. Sample Collection and DNA Extraction

In April and May 2025, parasitic ticks were collected from domestic animals in Menyuan County in Haibei Tibetan Autonomous Prefecture, Qinghai Province, northwest China. All animals in the sheepfolds in the sampling location, including nine free-ranging goats (*Capra hircus*) and four free-ranging sheep (*Ovis aries*), were manually restrained and examined. Half-body tick collections were made. All the ticks were carefully removed from the body (ear, face, groin, etc.) of domestic animals using tweezers. A total of 136 ticks were collected. Of those, 90 ticks were collected from goats, and 46 ticks were from sheep. After morphological identification by observing the basis capitula, anal groove, cervical groove, and palp [10], the tick species was first determined and then washed using Phosphate-Buffered Saline (PBS) (Thermo Scientific, Waltham, MA, USA) before DNA extraction to exclude contamination. The ticks were individually ground into homogenate with PBS (100 μL) in a mortar. The DNA was extracted using Omega Mollusc DNA extraction kits (Omega Bio-Tek, Norcross, GA, USA). ddH_2_O was used as a negative control. The DNA was eluted in 100 μL elution buffer. The concentration and quality of the DNA were measured using a NanoDrop 2000 spectrophotometer (Thermo Scientific, Waltham, MA, USA). The DNA was stored in a −80 °C refrigerator before molecular detection of the pathogens. The tick species were molecularly confirmed by amplifying and analyzing the *COI* gene using primers [11].

### 2.2. Molecular Identification of Brucella spp., Coxiella sp., and Theileria/Babesia

All the DNA samples were screened for *Brucella* spp. by semi-nested PCR amplifying a unique repeat sequence on chromosome 1 of *Brucella* spp. according to reference [12], generating approximately 310–330 bp PCR products. For further identification of the *Brucella* species, partial sequences of the *omp25* (635 bp), *bcsp31* (737 bp), and *rpoB* (424 bp) genes, which were usually used for *Brucella* species identification, were obtained by nested PCR and sequencing (primers shown in references Li et al. [13], Li et al. [14], and Table 1). PCR was performed using 2× Easy Taq PCR Supermix (TransGen Biotech, Beijing, China) in a SensoQuest Labcycler (40 cycles) (SensoQuest, Göttingen, Germany). The DNA of *B. melitensis* 16M was set as a positive control and ddH_2_O as a negative control.

*Coxiella* sp. was detected using a set of nested primers amplifying a partial sequence of the *rpoB* gene, yielding approximately 500 bp PCR products (primers shown in Duron et al. [14]). The DNA of *C. burnetii* was set as a positive control and ddH_2_O was set as a negative control. For confirmation and further identification, the partial *DnaK* sequence of detected strains were recovered using nested primers [17]. Protozoan (mainly *Theileria* and *Babesia*) pathogens were detected using nested primers amplifying the 18S sequence (approximately 700 bp) (primers shown in Zhang et al. [18]). The DNA of *Babesia orientalis* was set as a positive control and ddH_2_O was set as a negative control. All the PCR products were electrophoresed in 1.0% agarose gels, and the PCR products that met the expected length were subjected to Sanger sequencing.

### 2.3. Phylogenetic Analysis

All the recovered sequences were assembled and manually edited using BioEdit software (Ver7.2.5). The recovered sequences were aligned with reference sequences in the GenBank Database using BLASTn (https://blast.ncbi.nlm.nih.gov/Blast.cgi (accessed on 4 January 2026)) to determine the species of the detected bacteria or protozoan as well as the nucleotide similarities. For phylogenetic analysis, all the recovered sequences were locally aligned with downloaded reference sequences using the ClustalW method in the MEGA program [19]. Maximum Likelihood (ML) trees were constructed in the GTR model using PhyML based on the aligned DNA sequences [20]. All the phylogenetic trees were mid-point rooted.

## 3. Results

### 3.1. Collection of Tick Samples

**Figure 1:**
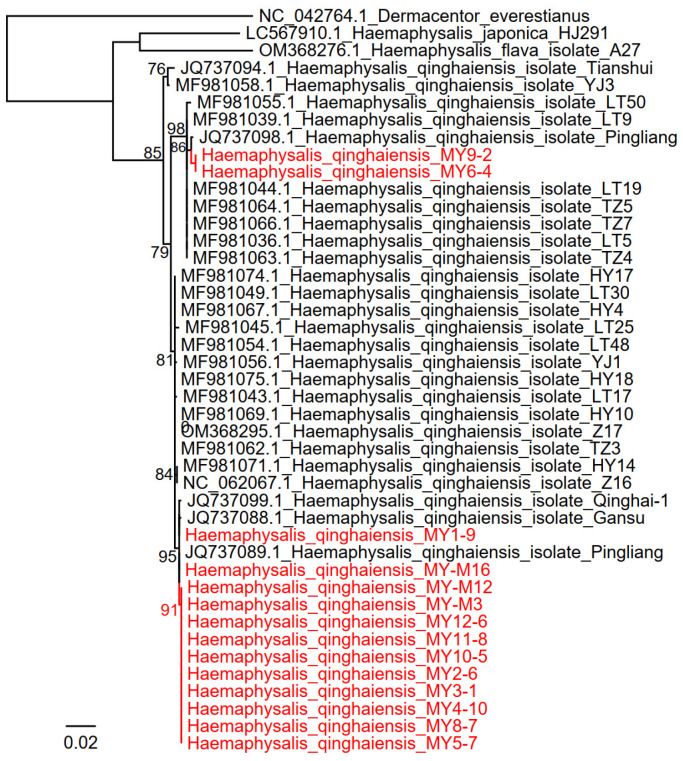
Phylogenetic trees based on the *COI* sequences of *Haemaphysalis qinghaiensis* ticks from Menyuan County in Qinghai Province, China.

All the 136 ticks were morphologically identified to be *H. qinghaiensis* by observing the basis capitula, anal groove, cervical groove, and palp. All these 136 ticks were fully or partially engorged. The tick species were confirmed by PCR amplifying and sequencing the partial Cytochrome coxidase subunit I (*COI*) gene (691 bp) of randomly selected ticks. All the obtained *COI* gene sequences have >99% identities to *H. qinghaiensis*, confirming that all these 136 ticks are *H. qinghaiensis*. Phylogenetic analysis of the *COI* genes showed that the ticks formed three clades in the phylogenetic tree, indicating the high genetic diversity of *H. qinghaiensis* in Qinghai Province (Figure 1).

### 3.2. Detection and Analysis of the Brucella spp.

**Figure 2:**
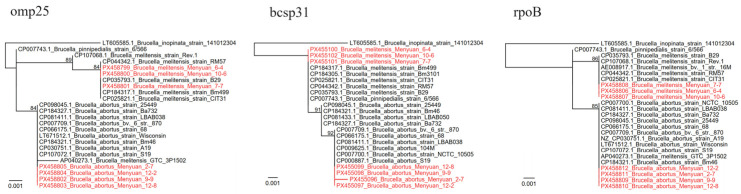
Phylogenetic trees based on the nucleotide sequences of the *omp25*, *bcsp31*, and *rpoB* genes of *Brucella* strains.

PCR results showed that two *Brucella* species were identified in *H. qinghaiensis* ticks: *B. melitensis* and *B. abortus*, with positive rates of 4.41% (6/136) and 3.68% (5/136), respectively (Table 1). Notably, the samples that were positive for *B. melitensis* and *B. abortus* were distributed in ticks collected from both goats and sheep. For further identification, three key genes of *Brucella* spp., the *bcsp31*, *rpoB*, and *omp25* genes, were recovered and analyzed from seven randomly selected samples. All the *bcsp31*, *rpoB*, and *omp25* sequences of *B. melitensis* were 100% identical to those of *B. melitensis* strain B29 (CP035793.1, from China), *B. melitensis* strain RM57 (CP044342.1, from China), *B. melitensis* strain BM499 (CP184317.1, from Kazakhstan), etc. For the *B. abortus* strains, their *omp25* and *rpoB* sequences were both 100% identical to the *B. abortus*_bv. 6 str. 870 (CP007709.1, from the USA), *B. abortus* strain 25,449 (CP098045.1, from Italy), *B. abortus* strain LBAB038 (CP081411.1, from Brazil), and *B. abortus* strain S19 (CP107072.1, from China), etc. (Figure 2). Interestingly, for the *bcsp31* gene, there are some differences. The sequences of *B. abortus* Menyuan-9-9, *B. abortus* Menyuan-12-2, and *B. abortus* Menyuan-12-8 are also 100% identical to these above reference strains, while the sequence of *B. abortus* Menyuan-2-7 is only 99.86% identical to the above strains. In the phylogenetic tree, it also forms a different branch (Figure 2).

### 3.3. Detection and Analysis of the Coxiella

*Coxiella* was detected in *H. qinghaiensis* with an extremely high positive rate of 97.06% (132/136) (Table 2). The *rpoB* sequences from the randomly selected strains were 100% identical or had one different nucleotide (99.79%) from each other. BLASTN showed that they have highest similarity, 99.16–99.37%, to the uncultured *Coxiella* sp. clone tick166 (OK625735.1) and the uncultured *Coxiella* sp. clone tick103 (OK625734.1), both of which were from *H. qinghaiensis* ticks in Ngawa Prefecture, Sichuan Province, China. In the phylogenetic tree, they are divided into two closely related clades. Interestingly, all the *DnaK* sequences were identical, and they are only 97.61% identical to the *Coxiella* endosymbiont of *Dermacentor silvarum* isolate Dsilv1 (KP985401.1) and 97.45% identical to the *Coxiella* endosymbiont of *Haemaphysalis flava*. In both phylogenetic trees, the detected strains were closely related to the *Coxiella* endosymbiont of ticks but distant from the human pathogenic *C. burnetii*. Therefore, we suppose that all the strains are the *Coxiella* endosymbiont of *H. qinghaiensis*.

### 3.4. Detection and Analysis of the Theileria/Babesia

Two protozoan species were identified in the ticks. Both of them belong to the genus *Theileria*: *T. luwenshuni* and *T. uilenbergi*. They were detected in ticks collected from both goats and sheep. *Theileria luwenshuni* was detected with a higher positive rate of 75.00% (102/136), while *T. uilenbergi* was detected in 16.18% (22/136) of the ticks. Genetic and phylogenetic analysis indicated that all the *T. luwenshuni* sequences were identical to each other, and they were 100% identical to *T. luwenshuni* strains (JF719832.1, JX469527.1, MH179336.1, etc.) previously detected in ticks or small ruminants from the Sichuan, Qinghai, and Gansu provinces in China. Similarly, all the DNA sequences of *T. uilenbergi* strains were identical to each other, and they were 100% identical to voles, goats, or ticks from China.

All the obtained sequences have been submitted to the GenBank Database (accession numbers shown in Table S1).

## 4. Discussion

*Haemaphysalis qinghaiensis* is an endemic tick species distributed in the western plateau areas of China, such as in the Qinghai, Gansu, Sichuan, and Yunnan provinces [4]. It has a wide range of hosts, including goats, sheep, cattle, yaks, and horses. As a three-host tick, *H. qinghaiensis* changes its host during its development and it also occasionally bites humans [5]. However, compared to other tick species, the human and animal pathogens carried by *H. qinghaiensis* are still rarely studied.

Brucellosis caused by *Brucella* spp. is considered one of the most common zoonotic diseases worldwide and it has been designated as one of the world’s most important “neglected zoonotic diseases” by the World Health Organization (WHO) [21,22]. Numerous humans and animals have been infected, with an estimated 2,100,000 human cases annually worldwide [23]. The infection of humans with brucellosis usually occurs due to direct or indirect inhalation of infected animals’ infectious materials, or consumption of unpasteurized animal products (milk, meat, etc.) [24]. Although the role of ticks in the transmission of brucellosis is still unclear, *B. melitensis* and *B. abortus* have been detected in the eggs and larvae of *Dermacentor marginatus*, suggesting the transovarial transmission of *Brucella* spp. in *D. marginatus* ticks [25]. Some other studies also reported the presence of *Brucella* spp. in various tick species, including *Haemaphysalis longicornis*, *Ornithodoros lahorensis*, etc. [26,27,28]. However, *Brucella* spp. have never been reported in *H. qinghaiensis* ticks. This may be the first report that shows that *H. qinghaiensis* harbors *Brucella* spp. Qinghai Province is an endemic area of brucellosis. *Brucella melitensis*, *B. abortus*, and *B. suis* have been reported in domestic and wild animals in Qinghai [29]. In this study, although we identified *B. melitensis* and *B. abortus* in ticks (Table 2), it is possible that the DNA of *Brucella* was from the blood of sheep or goats due to the fact that the ticks were collected from them and were engorged. Whether *H. qinghaiensis* ticks play a role in the maintenance and transmission of *Brucella* spp. is still to be determined.

**Figure 3:**
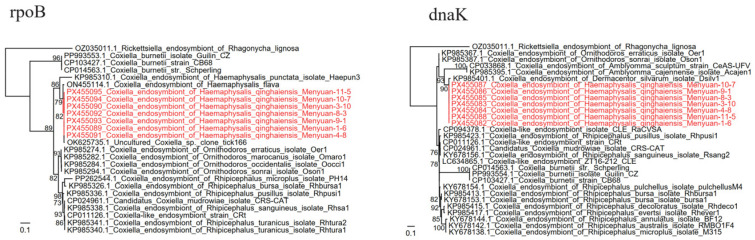
Phylogenetic trees based on the nucleotide sequences of the *rpoB*, and *dnaK* genes of *Coxiella* strains.

*Coxiella burnetii*, the agent of Q fever, is an important zoonotic pathogen with a worldwide distribution. Ticks have been proven to be the hosts and vectors of *C. burnetii*. In this study, we tried to detect *C. burnetii* in ticks from Qinghai Province. However, the results indicated that *H. qinghaiensis* ticks have an extremely high positive rate of the *Coxiella* endosymbiont instead of *C. burnetii*. Although some studies reported the pathogenicity of certain *Coxiella* endosymbionts in animals [15], most of the *Coxiella* endosymbionts of ticks were considered non-pathogenic. Furthermore, the *Coxiella* endosymbiont of *H. qinghaiensis* strains are genetically distant from pathogenic *C. burnetiid* (Figure 3), which also suggests its non-pathogenicity. This result indicates that this area may not be an endemic area with Q fever.

**Figure 4:**
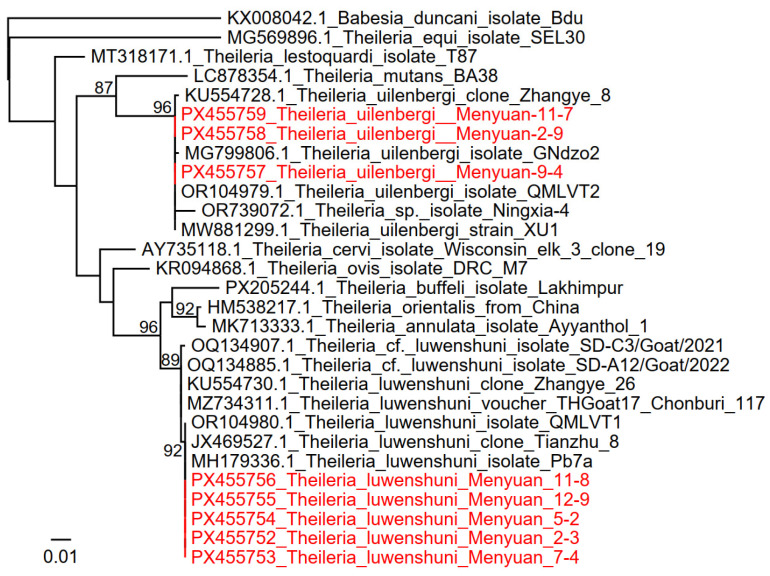
Phylogenetic trees based on the nucleotide sequences of *COI* genes of *Theileria* strains.

*Theileria* and *Babesia* (usually called piroplasmids) are parasitic protozoa belonging to the class Piroplasmea, order Piroplasmorida [16]. They are tick-borne pathogens which infect multiple domestic and wild animals, causing piroplasmosis. Infected animals usually present with fever, anemia, malaise, lethargy, and anorexia [30]. In China, various piroplasmids have been reported in ticks and animals, including *B. bigemina*, *B. bovis*, *T. equi*, *T. annulata*, *T. orientalis*, etc. [18,31,32]. These piroplasmids cause significant economic losses in animal husbandry and have a significant effect on veterinary medicine. Occasionally, some of them (mainly *Babesia* spp.) may infect humans [33]. In this study, we reported *T. luwenshuni* and *T. uilenbergi* in ticks from Qinghai with high positive rates (Table 2, Figure 4). Our results may reflect the high prevalence of *Theileria* spp. in goats and sheep in this area. Although the animals from which the ticks were collected appeared to be healthy, they may act as infection sources of piroplasmosis. Control measures should be taken to prevent their spreading. Moreover, as recent as in 2025, *T. luwenshuni* was reported to infect humans, with 13 patients diagnosed in Yunnan Province, China [9]. This report suggests that *Theileria* might be a neglected or underestimated zoonotic pathogen. Due to the high positive rate of *Theileria* in ticks from this area, surveillance in local populations with relative symptoms is necessary.

There are some limitations in this study. First, all ticks were collected from domestic animals. This means that some microorganisms may not be maintained by the ticks but are just from their blood meals. Second, the sampling location is in only one county. Therefore, the results may not be representative of Qinghai Province. In future studies, surveillance in free-living ticks from more locations are needed.

## 5. Conclusions

In conclusion, we identified *Brucella melitensis*, *Brucella abortus*, the *Coxiella* endosymbiont of *Haemaphysalis qinghaiensis*, *Theileria luwenshuni* and *Theileria uilenbergi* in *Haemaphysalis qinghaiensis* ticks from Qinghai Province. Our results indicated that *H. qinghaiensis* harbors an extensive diversity of important zoonotic pathogens, and it may play important role in te maintenance and transmission of these pathogens. More surveillance is needed to determine their circulation in domestic animals and local populations.

## Figures and Tables

**Table 1:** The primers used for molecular detection and identification of the *Brucella* strains by hemi-nested PCR.

Primer Name	Targeted Gene	Round	Sequence	Annealing Temperature	Amplicon Length	Reference
BruRbin5-Forward	*rpoB*	1, 2	5-CGAGTTCGATTCCAAGGACATCG-3	55 °C	450 bp	[15]
BruRbex3-Reverse	*rpoB*	1	5-ATATTGACATGGTCGATATCGAGAAC-3	55 °C		
BruRbin3-Reverse	*rpoB*	2	5-AACCTTTTCATCGATTTCGTCACC-3	55 °C		
Osong-F234-Forward	A unique repeat sequence	1, 2	5-ACTGCATGGCATTTTTCGCCC-3	53 °C	320–340 bp	[16]
Osong-R609-Reverse	A unique repeat sequence	1	5-GGGAAGAGCGTTACAGGCGT-3	53 °C		
Osong-inR-Reverse	A unique repeat sequence	2	5-CGCAAAGTGACGCCACAGAG -3	53 °C		
Bcspex5-Forward	*Bcsp31*	1	5-ATGACCTGGCATTCTTCACATC-3	53 °C	800 bp	[15]
Bcspin5-Forward	*Bcsp31*	2	5-CTGCGTTTTTAATCGTTTCAGTC-3	53 °C		
Bcsp3-Reverse	*Bcsp31*	1, 2	5-AGATCGGAACGAGCGAAATA-3	53 °C		

**Table 2:** Positive rates of detected pathogens in *H. qinghaiensis* in Qinghai Province.

Species		Goat Ticks	Sheep Ticks	Total
*Coxiella*	*Coxiella* endosymbiont of *H. qinghaiensis*	88/90 (97.78%)	44/46 (95.65%)	132/136 (97.06%)
*Brucella*	*Brucella melitensis*	5/90 (5.56%)	1/46 (2.17%)	6/136 (4.41%)
	*Brucella* *abortus*	3/90 (3.33%)	2/46 (4.35%)	5/136 (3.68%)
*Theileria*	*Theileria luwenshuni*	79/90 (87.78%)	23/46 (50.00%)	102/136 (75.00%)
	*Theileria uilenbergi*	6/90 (6.67%)	16/46 (34.78%)	22/136 (16.18%)

## Data Availability

The original data presented in the study are openly available in the GenBank Database (accession numbers shown in Table S1).

## Supplementary Materials

The following supporting information can be downloaded at https://www.mdpi.com/article/10.3390/tropicalmed11010017/s1, Table S1. Accession numbers of the obtained sequences of *Brucella*, * Coxiella* and *Theileria* strains in this study in the GenBank Database; Table S2. The primers used for molecular detection and identification of the *Brucella* strains by hemi-nested PCR.
