# Supplementary material for: Brucella, Coxiella, and Theileria Species DNA in Haemaphysalis qinghaiensis Ticks Collected from Goats and Sheep in Qinghai Province, Northwest China
Source: Trop Med Infect Dis. 2026 Jan 7;11(1):17. doi: 10.3390/tropicalmed11010017 (PMC12846645; doi:10.3390/tropicalmed11010017)
Supplement: Supplementary file 1 [file tropicalmed-11-00017-s001.zip › Table_S1.pdf]

Table S1. Accession numbers of the obtained sequences of *Brucella, Coxiella* and *Theileria* strains in this study in the GenBank Database.

| No. | **Gene** | **Genbank Number** | **Bacterial strain** |
|---|---|---|---|
| 1 | 18S | PX455752 | *Theileria_luwenshuni_*Menyuan-2-3 |
| 2 | 18S | PX455753 | *Theileria_luwenshuni_* Menyuan-7-4 |
| 3 | 18S | PX455754 | *Theileria_luwenshuni_* Menyuan-5-2 |
| 4 | 18S | PX455755 | *Theileria_luwenshuni_* Menyuan-12-9 |
| 5 | 18S | PX455756 | *Theileria_luwenshuni_* Menyuan-11-8 |
| 6 | 18S | PX455757 | *Theileria_uilenbergi_* Menyuan-9-4 |
| 7 | 18S | PX455758 | *Theileria_uilenbergi_* Menyuan-2-9 |
| 8 | 18S | PX455759 | *Theileria_uilenbergi_* Menyuan-11-7 |
| 9 | *dnaK* | PX455082 | *Coxiella_endosymbiont_*of*_Haemaphysalis_qinghaiensis_*Menyuan-1-6 |
| 10 | *dnaK* | PX455083 | *Coxiella_endosymbiont_*of*_Haemaphysalis_qinghaiensis_*Menyuan-3-10 |
| 11 | *dnaK* | PX455084 | *Coxiella_endosymbiont_*of*_Haemaphysalis_qinghaiensis_*Menyuan-4-8 |
| 12 | *dnaK* | PX455085 | *Coxiella_endosymbiont_*of*_Haemaphysalis_qinghaiensis_*Menyuan-8-3 |
| 13 | *dnaK* | PX455086 | *Coxiella_endosymbiont_*of*_Haemaphysalis_qinghaiensis_*Menyuan-9-1 |
| 14 | *dnaK* | PX455087 | *Coxiella_endosymbiont_*of*_Haemaphysalis_qinghaiensis_*Menyuan-10-7 |
| 15 | *dnaK* | PX455088 | *Coxiella_endosymbiont_*of*_Haemaphysalis_qinghaiensis_*Menyuan-11-5 |
| 16 | *rpoB* | PX455089 | *Coxiella_endosymbiont_*of*_Haemaphysalis_qinghaiensis_*Menyuan-1-6 |
| 17 | *rpoB* | PX455090 | *Coxiella_endosymbiont_*of*_Haemaphysalis_qinghaiensis_*Menyuan-3-10 |
| 18 | *rpoB* | PX455091 | *Coxiella_endosymbiont_*of*_Haemaphysalis_qinghaiensis_*Menyuan-4-8 |
| 19 | *rpoB* | PX455092 | *Coxiella_endosymbiont_*of*_Haemaphysalis_qinghaiensis_*Menyuan-8-3 |
| 20 | *rpoB* | PX455093 | *Coxiella_endosymbiont_*of*_Haemaphysalis_qinghaiensis_*Menyuan-9-1 |
| 21 | *rpoB* | PX455094 | *Coxiella_endosymbiont_*of*_Haemaphysalis_qinghaiensis_*Menyuan-10-7 |
| 22 | *rpoB* | PX455095 | *Coxiella_endosymbiont_*of*_Haemaphysalis_qinghaiensis_*Menyuan-11-5 |
| 23 | *Bcsp31* | PX455096 | *Brucella_abortus_* Menyuan-2-7 |
| 24 | *Bcsp31* | PX455097 | *Brucella_abortus_* Menyuan-12-2 |
| 25 | *Bcsp31* | PX455098 | *Brucella_abortus_* Menyuan-9-9 |
| 26 | *Bcsp31* | PX455099 | *Brucella_abortus_* Menyuan-12-8 |
| 27 | *Bcsp31* | PX455100 | *Brucella_melitensis_*Menyuan-6-4 |
| 28 | *Bcsp31* | PX455101 | *Brucella_melitensis_*Menyuan-7-7 |
| 29 | *Bcsp31* | PX455102 | *Brucella_melitensis_*Menyuan-10-6 |
| 30 | *Omp25* | PX458805 | *Brucella_abortus_* Menyuan-2-7 |
| 31 | *Omp25* | PX458804 | *Brucella_abortus_* Menyuan-12-2 |
| 32 | *Omp25* | PX458802 | *Brucella_abortus_* Menyuan-9-9 |
| 33 | *Omp25* | PX458803 | *Brucella_abortus_* Menyuan-12-8 |
| 34 | *Omp25* | PX458799 | *Brucella_melitensis_*Menyuan-6-4 |
| 35 | *Omp25* | PX458801 | *Brucella_melitensis_*Menyuan-7-7 |
| 36 | *Omp25* | PX458800 | *Brucella_melitensis_*Menyuan-10-6 |
| 37 | *rpoB* | PX458811 | *Brucella_abortus_* Menyuan-2-7 |

| | | | |
|---|---|---|---|
| 38 | *rpoB* | PX458812 | *Brucella_abortus_* Menyuan-12-2 |
| 39 | *rpoB* | PX458809 | *Brucella_abortus_* Menyuan-9-9 |
| 40 | *rpoB* | PX458810 | *Brucella_abortus_* Menyuan-12-8 |
| 41 | *rpoB* | PX458806 | *Brucella_melitensis*_Menyuan-6-4 |
| 42 | *rpoB* | PX458808 | *Brucella_melitensis*_Menyuan-7-7 |
| 43 | rpoB | PX458807 | *Brucella_melitensis*_Menyuan-10-6 |
