# Supplementary material for: Brucella, Coxiella, and Theileria Species DNA in Haemaphysalis qinghaiensis Ticks Collected from Goats and Sheep in Qinghai Province, Northwest China
Source: Trop Med Infect Dis. 2026 Jan 7;11(1):17. doi: 10.3390/tropicalmed11010017 (PMC12846645; doi:10.3390/tropicalmed11010017)
Supplement: Supplementary file 1 [file tropicalmed-11-00017-s001.zip › Table_S2.pdf]

Table S2. The primers used for molecular detection and identification of the *Brucella* strains by hemi-nested PCR.

Primer	Gene	Cycle	Sequence	Anticipated amplicon length
BruRbin5	*rpoB*	1, 2	5- CGAGTTCGATTCCAAGGACATCG-3	450 bp
BruRbex3	*rpoB*	1	5-ATATTGACATGGTCGATATCGAGAAC-3	
BruRbin3	*rpoB*	2	5-AACCTTTTCATCGATTTCGTCACC-3	
Osong-F234	A unique repeat sequence	1, 2	5-ACTGCATGGCATTTTTCGCCC-3	320-340 bp
Osong-R609	A unique repeat sequence	1	5-GGGAAGAGCGTTACAGGCGT-3	
Osong-inR	A unique repeat sequence	2	5-CGCAAAGTGACGCCACAGAG -3	
Bcspex5	*Bcsp31*	1	5-ATGACCTGGCATTCTTCACATC-3	800 bp
Bcspin5	*Bcsp31*	2	5-CTGCGTTTTTAATCGTTTCAGTC-3	
Bcsp3	*Bcsp31*	1, 2	5-AGATCGGAACGAGCGAAATA-3	
